# Endoscopic injection sclerotherapy for treating recurrent bleeding of small bowel angioectasias

**DOI:** 10.1186/s12876-023-02836-x

**Published:** 2023-07-11

**Authors:** Jing Yang, Lei Zhou, Dan Xu, Yan Fan, Heng Zhang

**Affiliations:** Department of Gastroenterology, The Central Hospital of Wuhan, Tongji Medical College, Huazhong University of Science and Technology, No.26, Shengli Street, Jiang’an District, Wuhan, Hubei Province 430014 China

**Keywords:** Small bowel angioectasias, Double-balloon enteroscopy, Capsule endoscopy, Endoscopic injection sclerotherapy

## Abstract

**Background:**

There is still no consensus on the preferred endoscopic therapy for small bowel angioectasias (SBAs). The aim of this study was to evaluate effectiveness and safety of endoscopic injection sclerotherapy (EIS) for treating recurrent bleeding of SBAs.

**Methods:**

Sixty-six adult patients diagnosed with SBAs by capsule endoscopy (CE) or double-balloon enterscopy (DBE) examinations were enrolled in this retrospective study from September 2013 to September 2021. The patients were divided into an EIS group (35 cases) and a control group (31 cases) according to whether they underwent EIS treatment. Clinical characteristics, medical histories, lesion characteristics, main laboratory indicators, treatments, and outcomes were collected. The rates of re-bleeding, re-admission, and red blood cell (RBC) transfusion were compared between different groups after discharge. The rates of hospitalization and RBC transfusion were compared between before admission and after discharge in both groups. Odds ratios (ORs) and 95% confidence intervals (CIs) were used in the multivariate logistic regression analysis to assess relative factors for re-bleeding.

**Results:**

All the rates of re-bleeding, re-admission and RBC transfusion after discharge in the EIS group were significantly lower than those in the control group (all *P* < 0.05). The rates of hospitalization and RBC transfusion after discharge were significantly lower than those before admission in the EIS group (both *P* < 0.05), while those did not reach significant differences in the control group (both *P* > 0.05). Multivariate logistic regression analysis showed that RBC transfusion before admission (OR, 5.655; 95% CI, 1.007–31.758, *P* = 0.049) and multiple lesions (≥ 3) (OR, 17.672; 95% CI, 2.246–139.060, *P* = 0.006) were significant risk factors of re-bleeding, while EIS treatment (OR, 0.037; 95% CI, 0.005–0.260, *P* < 0.001) was a significant protective factor. No endoscopic adverse events were observed during hospitalization and none of the enrolled patients died within 12 months after discharge.

**Conclusion:**

EIS treatment had good effectiveness and safety for treating recurrent bleeding of SBAs, which could be considered as one of the first-line endoscopic treatment options for SBAs.

## Introduction

Small bowel bleeding (SBB) accounts for 5–10% of all patients presenting with gastrointestinal bleeding [1]. Patients with SBB can have recurrent episodes of bleeding and require multiple hospital admissions and frequent blood transfusion [2]. Small bowel vascular lesions, particularly angioectasias, are the most common causes of SBB [3, 4]. Despite the increasing application of endoscopic therapies under balloon-assisted enteroscopy (BAE), high recurrence rates still present in these diseases [5, 6]. There were several therapeutic approaches for small bowel angioectasias (SBAs), while no consensus on best endoscopic therapy of SBAs was achieved [6].

Argon plasma coagulation (APC), a kind of noncontact thermal therapy, is the conventional method of endoscopic therapy for SBAs [6]. However, the effectiveness of endoscopic methods including this technique for treating SBAs is controversial. A previous systemic review showed comparative re-bleeding rates between patients underwent endoscopic treatment and those received no therapy [7]. Samaha, et al. [8] reported that the re-bleeding rate was 46% (45/98) at 36 months in the patients diagnosed as small bowel vascular lesions and treated using mainly APC. Ponte, et al. [9] conducted a retrospective double-center investigation of patients with SBAs undergoing a second enteroscopy treatment (using mainly APC) due to a first re-bleeding episode; and the results suggested that most re-bleeding episodes occurred within the first 12 months of follow-up, resulting in a re-bleeding rate of 33.1% at 6 months, 39.1% at 12 months and 52.6% at 24 months. The guideline of American College of Gastroenterology also stated that data on endoscopic therapy for SBAs is limited and its effectiveness has not been determined [1]. Furthermore, considering the low thickness of small bowel wall, APC should be performed very prudently and carefully for SBAs, particularly for multiple lesions and suspected lesions. The technical review of European Society of Gastrointestinal Endoscopy (ESGE) suggested that pre-injection of saline into the submucosa should be performed before application of APC for small bowel vascular lesions [10]. Thus, performing multi-focal APC therapy in small bowel without submucosal injection may be dangerous. On the contrary, frequent submucosal injections and instrument alternations may be very time-consuming. These facts push the performers of BAE examinations to pursue endoscopic methods more maneuverable, time-saving, secure and effective for treating SBAs.

Endoscopic injection sclerotherapy (EIS), a method usually used as a treatment for esophageal varices [11], has been used to treat patients with various gastrointestinal vascular lesions, such as gastric antral vascular ectasia [12], rectal varices [13], ectopic varices [14], small bowel hemangiomas [15], vascular malformation in blue rubber bleb nevus syndrome [16], and even small bowel huge hemolymphangioma [17]. The mechanisms of this method may be pressure on blood vessels associated with interstitial edema, thrombus formation, and secondary vascular inflammation [6]. A study from Japan used polidocanol injection for the treatment of SBAs and reported a relatively low re-bleeding rate (7/53) [18]. However, recurrent bleeding of SBAs is still a challenging clinic problem. There are few data to evaluate EIS for SBAs among the Chinese population and efficiency of EIS for treating recurrent bleeding of SBAs. Therefore, the aim of this study was to evaluate effectiveness and safety of EIS for treating recurrent bleeding of SBAs.

## Patients and methods

### Study design

This study was designed as a retrospective study. The study followed the tenets of the Declaration of Helsinki and was approved by the Ethics Committee of The Central Hospital of Wuhan (approve number: No. 2016-12). Written informed consent was obtained from all the subjects.

### Patients

**Fig. 1:**
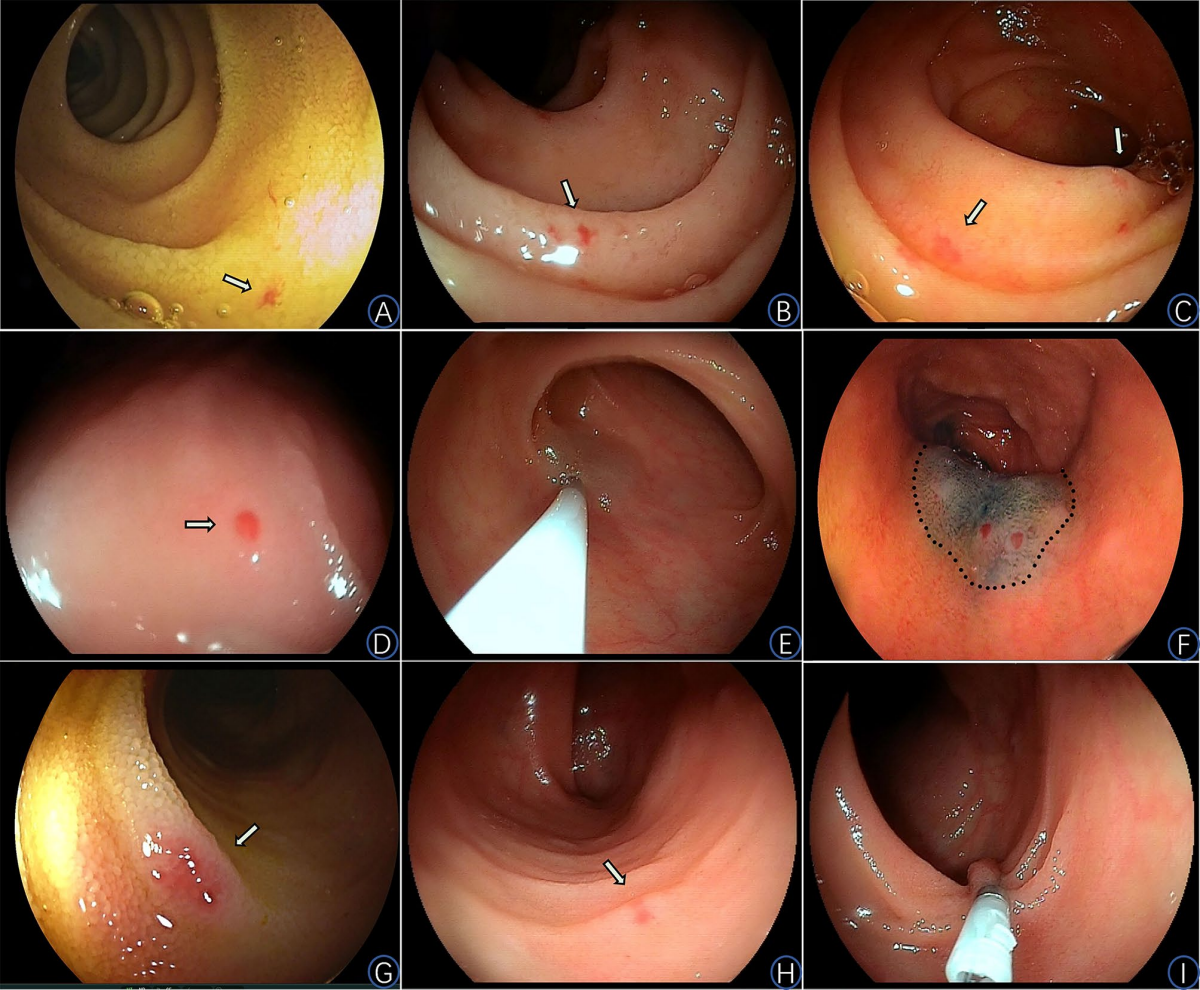
Small bowel angioectasias and corresponding endoscopic treatments **A-D**: Type 1a lesions with or without oozing **E**: The local mucosa was lifted and whitened by the submucosal injection with sclerosing agent **F**: A endoscopic image showing the range of endoscopic treatment, the local mucosa was lifted by the submucosal injection with a mixture of 1% lauromacrogol combined with 0.5% methylene blue solution **G-H**: Type 1b lesions without oozing **I**: A endoscopic image showing a type 1b lesion treated by a hemostatic clip prior to EIS treatment

Consecutive adult patients who were admitted in department of gastroenterology of The Central Hospital of Wuhan and diagnosed with SBAs by capsule endoscopy (CE, MiroCam MC1000, IntroMedic Co., Ltd, Korea) or double-balloon enteroscopy (DBE, one kind of BAE, Fujinon EN-450T5, Fujinon Inc., Japan) between September 2013 and September 2021 were enrolled. Diagnostic criteria of SBAs were as follows: (1) melena and/or hematochezia; (2) positive fecal occult blood; (3) clear pictures or videos of SBAs were captured; (4) no other causes of bleeding confirmed by examinations. According to Yano-Yamamoto classification [16], SBAs are classified into type 1 lesions. Type 1a lesions (Fig. 1A to D) are characterized by punctate erythema (< 1 mm) with or without oozing, and type 1b lesions (Fig. 1G and H) are characterized by patchy erythema (2–3 mm) with or without oozing.

Inclusion criteria were as follows: (1) entire visualization of small bowel by CE or DBE; (2) bleeding presented at least twice in 12 months before admission; (3) age ≥ 18 and ≤ 85 years old; (4) the follow-up time was ≥ 12 months. Exclusion criteria were as follows: (1) patients who suffered bleeding at the first time; (2) patients with long-term use of hormones or non-steroidal anti-inflammatory drugs; (3) patients with severe esophageal and/or gastric varices; (4) patients with severe coagulation dysfunction; (5) the follow-up time was < 12 months.

The patients were divided into the EIS group and the control group according to whether they underwent EIS treatment. The patients who only underwent CE examinations and refused further DBE procedures were assigned to the control group. The patients who underwent CE examinations and subsequent endoscopic treatments (antegrade and/or retrograde DBE), or directly underwent DBE examinations (antegrade and retrograde) and synchronous EIS were assigned to the EIS group.

### CE and BAE procedures

Prior to DBE and CE, esophagogastroduodenoscopy and colonoscopy with terminal ileoscopy were performed in all patients at least once. Meanwhile, computed tomography enterography was performed in hemodynamically stable patients, and computed tomography angiography was performed in those with brisk active bleeding.

Three experienced endoscopists (Y.J., Z.L. and X.D.) took charge of all CE and DBE procedures. All patients gave written informed consent before CE or DBE. They were instructed to consume clear liquid diet at least 1 day and insist overnight fasting (8–12 h) before the CE or DBE procedures. The patients were orally administered 2000 mL and 30 mL of polyethylene-glycol solution and simethicone emulsion, respectively, for bowel preparation on the morning of CE examinations or retrograde DBE examinations. The capsule was swallowed 4 h after the finish of bowel preparation. The sensors and recording device were removed 12 h after swallowing the capsule. Images and videos were analyzed subsequently. Similar to the preparation for upper gastrointestinal endoscopy, antegrade DBE required only a fast for 8–12 h. Retrograde DBE and antegrade DBE were performed separately. The initial approach was determined according to the patient’s clinical manifestation, and the position of abnormalities provided by other examinations prior to BAE. Hemostatic clips were employed to mark the end point of observation.

### Strategy of endoscopic treatment

DBE was inserted persistently until the visual field could not be further advanced or endoscopic docking was achieved. The small bowel mucosa was closely observed, and a water pump was employed to ensure a clear visual field. Type 1a lesions were treated with EIS, and type 1b lesions were treated with EIS or EIS combined with clipping (Fig. 1E, F and I). All responsible lesions or suspicious lesions were treated. When performing, 0.5-1.0 mL sclerosing agent (1% lauromacrogol, Shaanxi TIANYU Pharmaceutical Co., Ltd., China) was injected into each lesion at local submucosa until the local mucosa was lifted and whitened (Fig. 1E). The range should not be more than 1/2 of the circumferences of intestinal wall (Fig. 1F). For larger type 1b lesions, clipping was performed before EIS treatment and the points of injection were located around the hemostatic clip.

## Evaluation

Patients’ clinical characteristics including age, sex, history of smoking, history of drinking, drug use of antithrombotic, personal history of malignancy, history of gastrointestinal surgery and underlying diseases (hypertension, cardiovascular disease, chronic renal failure, diabetes, and liver cirrhosis) at this admission were recorded. In addition, the relevant medical histories including the frequency of bleeding occurrence, the volume of red blood cell (RBC) transfusion, and the times of hospitalization within 12 months before admission were collected and analyzed. Bleeding for many times within 1 week was considered as 1 time of bleeding occurrence. Lesion characteristics including type, location, and number, as well as main laboratory indicators including hemoglobin (HB), blood platelet (PLT), prothrombin time (PT), activated partial thromboplastin time (APTT), total bilirubin (TBIL), and serum albumin (ALB) were analyzed.

Among the current hospitalization, routine medical treatment according to the 2015 ACG clinical guideline [1], was performed in all patients. The patients with present bleeding, were treated with hemostatic drug (octreotide, Novartis Pharma AG, Switzerland), intravenous iron, transfusion of packed RBCs and so on. For those with yellow feces or negative results of fecal occult blood, intravenous iron and RBC transfusion were given according to the clinical needs. Patients were discharged when they met the following criteria: (1) with yellow feces or negative results of fecal occult blood; (2) HB levels were stable and > 70 g/L; (3) without anemia related severe symptoms. The post-endoscopic adverse events were recorded in detail. The lengths of stay (LOSs) were recorded and compared between groups.

All patients were followed up by outpatient or telephone interview at least once every 2 months after discharge, and the ranges of follow-up were at least 12 months. Blood routine and stool routine were reviewed to assess the patient’s condition. Iron was given orally for the patients with anemia after discharge. The patients were instructed to proactively seek medical attention when they suffered bleeding occurrence. Re-admissions were recommended when patients met one of the following conditions: (1) hematochezia without spontaneous remission; (2) melena for ≥ 3 days, with positive result of fecal occult blood, and without spontaneous remission; (3) a decrease in the HB level by > 20 g/L from baseline; (4) the HB level < 70 g/L; (5) with anemia-associated severe symptoms. Within 12 months after discharge, the frequency of bleeding occurrence, the volume of RBC transfusion, and the times of hospitalization were evaluated. Subsequently, the rates of re-bleeding, re-admission and RBC transfusion after discharge were evaluated and compared between groups. In addition, the rates of hospitalization and RBC transfusion after discharge were compared to those before admission in different groups.

### Statistical analysis

All analyses were conducted by using SAS program (version 9.4, SAS Institute Inc., Cary, NC, USA). The countable data was presented as mean ± *SD*. The measurement data were first tested for normality. While Student-t test was used for statistical analysis for the data conforming to normal distribution, nonparametric test was employed to statistically analyze the data that did not conform to normal distribution. The categorical variables were analyzed using chi-squared tests. Odds ratios (ORs) and 95% confidence intervals (CIs) were used in the multivariate logistic regression analysis to assess relative factors for re-bleeding. *P* value < 0.05 was considered statistically significant.

## Results

### Clinical information, endoscopic findings and main laboratory indicators of the enrolled patients

**Fig. 2:**
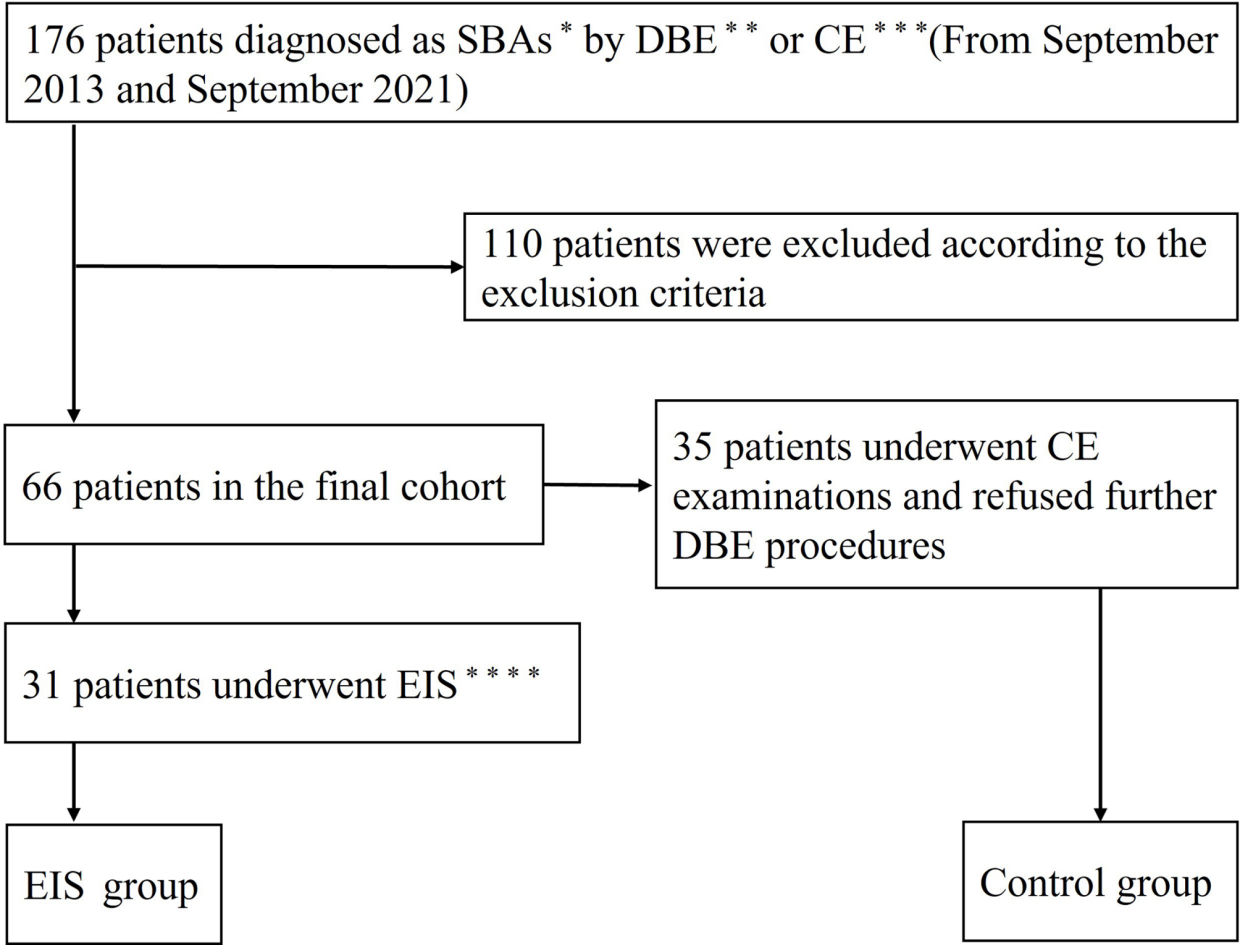
Flow diagram of patient inclusion in the study. A total of 176 patients diagnosed with SBAs by CE or DBE were primarily enrolled. One hundred and ten patients were excluded according to the exclusion criteria. Sixty-six patients were finally enrolled, who were divided into the EIS group and control group based on the treatments. ^*^Small bowel angioectasias; ^**^Double-balloon enteroscopy; ^***^Capsule endoscopy; ^****^Endoscopic injection sclerotherapy

A total of 66 patients were included in the final cohort. Thirty-five patients underwent CE examinations without further DBE procedures and were assigned to the control group (Fig. 2). Four patients underwent CE examinations with subsequent endoscopic treatments (antegrade and/or retrograde DBE) and 27 patients underwent DBE examinations (antegrade and retrograde) with synchronous EIS. A total of 31 patients were thus assigned to the EIS group (Fig. 2).

While the clinic information is summarized in Table 1, the endoscopic findings and main laboratory indicators of the enrolled patients are presented and analyzed in Table 2. The mean ages of the control group and EIS group were 65.86 ± 12.23 years old and 59.26 ± 7.96 years old with significant difference (*P* = 0.011). The percentage of female patients in the control group was significantly higher than that in the EIS group (65.71% versus 38.71%, *P* = 0.028). The mean of LOS in the control group was 9.49 ± 1.99 days, which was significantly higher than that in the EIS group (8.45 ± 1.31 days, *P* = 0.015). The percentages of patients with history of smoking, history of drinking, drug use of antithrombotic (mainly clopidogrel), personal history of malignancy, history of gastrointestinal surgery and bleeding-associated underlying diseases in the control group and in the SSB group were comparable (all *P* > 0.05, Table 1). In the total cohort, hypertension was the most common underlying disease, accounting for 63.64% (42/66), followed by liver cirrhosis, cardiovascular disease, diabetes and chronic renal failure, accounting for 12.12% (8/66, with two cases of alcoholic cirrhosis combined with hepatocellular carcinoma, two cases of posthepatitic cirrhosis, one case of schistosomiasis cirrhosis and one case of autoimmune cirrhosis in the EIS group, and with one case of posthepatitic cirrhosis and one case of schistosomiasis cirrhosis in the control group), 6.06% (4/66, with two cases of coronary heart disease and one case of valvulopathy in the EIS group, and with one case of coronary heart disease in the control group), 6.06% and 3.03% respectively. There were no patients with inflammatory bowel disease in the cohort. Before admission, all patients suffered 2–6 times bleeding occurrence and 74.24% of patients (49/66) had experienced at least one time of hospitalization; and most of them were referred from other medical centers without BAE equipment. For the first episode, bleeding went into spontaneous remission in the minority of patients, and the majority required hospitalization and hemostatic drugs (octreotide or somatostatin) to stop bleeding. None of the patients underwent enteroscopy treatment before the current hospitalization. 65.15% of patients (43/66) had experienced at least one time of RBC transfusion before the current admission. Between the two groups, the difference in the proportion of patients with hospitalization or RBC transfusion before admission was not significantly (either *P* > 0.05). Patients with type 1b lesions and patients with multiple lesions (≥ 3) were the majority in the total cohort, as well as in either group; and the proportion of patients with multiple lesions in the EIS group was significantly higher than that in the control group (93.55% versus 57.14%, *P* < 0.001). Lesions in the ileum were observed in 83.39% of patients (59/66), and the difference in the location of most lesions was not significant, *P* > 0.05. Lesions with active oozing/bleeding were captured in 2 patients of the control group and 5 patients of the EIS group. In addition, the means of main laboratory indicators in different groups were comparable (all *P* > 0.05, Table 2).

**Table 1:** Clinic information of patients in the study

Variables	Total cohort (*n* = 66)	Control group (*n* = 35)	EIS group (*n* = 31)	*P-*value
Age (years)				
Mean ± *SD*, range	62.76 ± 10.89, 42–85	65.86 ± 12.23, 44–85	59.26 ± 7.96, 42–74	0.011^*^
>65	23 (34.85)	18 (51.43)	5 (16.13)	0.003^*^
≤ 65	43 (65.15)	17 (48.57)	26 (83.87)	
Sex, *n* (%)				
Male	31 (46.97)	12 (34.29)	19 (61.29)	0.028^*^
Female	35 (53.03)	23 (65.71)	12 (38.71)	
History of smoking, *n* (%)	15 (22.73)	6 (17.14)	9 (29.03)	0.250
History of drinking, *n* (%)	8 (12.12)	2 (5.71)	6 (19.35)	0.188
Drug use of antithrombotic, *n* (%)	8 (12.12)	5 (14.29)	3 (9.68)	0.846
Personal history of malignancy, *n* (%)	5 (7.58)	1 (2.86)	4 (12.90)	0.283
History of gastrointestinal surgery, *n* (%)	6 (9.09)	4 (11.43)	2 (6.45)	0.785
Hospitalization before admission, *n* (%)	49 (74.24)	26 (74.29)	23 (74.19)	0.993
RBC transfusion before admission, *n* (%)	43 (65.15)	21 (60.00)	22 (70.97)	0.351
LOS (days)				
Mean ± *SD*	9.00 ± 1.77	9.49 ± 1.99	8.45 ± 1.31	0.015^*^
Median (range)	9 (6–14)	9 (6–14)	8 (6–11)	
**Underlying diseases**, ***n*(%)**				
Hypertension	42 (63.64)	22 (62.86)	20 (64.52)	0.889
Cardiovascular disease	4 (6.06)	1 (2.86)	3 (9.68)	0.521
Chronic renal failure	2 (3.03)	1 (2.86)	1 (3.23)	>0.999
Diabetes	4 (6.06)	0 (0.00)	4 (12.90)	0.094
Liver cirrhosis	8 (12.12)	2 (5.71)	6 (19.35)	0.188

NOTE: *: Compared the EIS group to the Control group, *P*<0.05

*Abbreviations*: *EIS*, endoscopic injection sclerotherapy; *SD*, standard deviation; *RBC*, red blood cell; *LOS*, length of stay

Variable definitions: History of smoking was defined as positive if the subject had smoked ≥ 5 cigarettes per day for ≥ 1 year and was still smoking or had quit within the previous 10 years. History of drinking was defined as positive if the subject’s alcohol consumption exceeded 50 g per day for ≥ 1 year. Drug use of antithrombotic was defined as positive if the patient had been taking antithrombotic for ≥ 2 weeks within 1 month prior to the current hospitalization

**Table 2:** Endoscopic findings and main laboratory indicators of patients in the study

Variables	Total cohort (*n* = 66)	Control group (*n* = 35)	EIS group (*n* = 31)	*P*-value
**Endoscopic findings,** ***n*** **(%)**				
Type of lesions				
Type 1a	18 (27.27)	10 (28.57)	8 (25.81)	0.801
Type 1b (with or without type 1a lesions)	48 (72.73)	25 (71.43)	23 (74.19)	
Location of most lesions				
Jejunum	7 (10.61)	1 (2.86)	6 (19.35)	0.076
Ileum	59 (89.39)	34 (97.14)	25 (80.65)	
Number of lesions				
< 3	17 (25.76)	15 (42.86)	2 (6.45)	<0.001^*^
≥ 3	49 (74.24)	20 (57.14)	29 (93.55)	
Active oozing/bleeding				
Yes	7 (10.61)	2 (5.71)	5 (16.13)	0.332
No	59 (89.39)	33 (94.29)	26 (83.87)	
**Main laboratory indicators, mean ±** ***SD***, **range**				
HB (g/L)	79.14 ± 21.52 46.00-140.00	77.83 ± 21.53 46.00-129.00	80.61 ± 21.76 47.00-140.00	0.604
PLT (10^9/L)	188.90 ± 87.44 33.00-426.00	178.70 ± 68.51 41.00-318.00	200.40 ± 104.80 33.00-426.00	0.330
PT (seconds)	11.37 ± 0.99 9.60–15.30	11.34 ± 1.08 9.60–15.30	11.41 ± 0.89 9.90–14.60	0.788
APTT (seconds)	26.07 ± 5.36 16.60–49.80	26.40 ± 6.35 16.60–49.80	25.71 ± 4.05 16.80–35.80	0.596
TBIL (umol/L)	10.83 ± 7.89 1.80–44.30	10.53 ± 6.79 2.80–42.90	11.17 ± 9.08 1.80–44.30	0.744
ALB (g/L)	36.20 ± 4.79 22.50–45.20	36.11 ± 5.47 22.50–45.20	36.30 ± 3.97 26.70–44.40	0.876

NOTE: *: Compared the EIS group to the Control group, *P*<0.05

*Abbreviations*: *EIS*, endoscopic injection sclerotherapy; *SD*, standard deviation; *HB*, hemoglobin; *PLT*, blood platelet; *PT*, prothrombin time; *APTT*, activated partial thromboplastin time; *TBIL*, total bilirubin; *ALB*, serum albumin

### Evaluation on the effectiveness of EIS treatment

The patients’ conditions after discharge in different groups are showed in Table 3. All the rates of re-bleeding, re-admission and RBC transfusion after discharge in the EIS group were significantly lower than those in the control group (all *P* < 0.05). In the control group, the rates of hospitalization and RBC transfusion after discharge were comparative to those before admission (both *P* > 0.05, Table 4). In contrast, the rates of hospitalization and RBC transfusion after discharge were significantly lower than those before admission in the EIS group (both *P* < 0.05, Table 4).

**Table 3:** Patients’ conditions after discharge in the EIS and control groups

Variables	Control group (*n* = 35)	EIS group (*n* = 31)	*P*-value
Re-bleeding, *n* (%)	21 (60.00)	7 (22.58)	0.002^*^
Re-admission, *n* (%)	21 (60.00)	4 (12.90)	< 0.001^*^
RBC transfusion, *n* (%)	14 (40.00)	3 (9.68)	0.005^*^

NOTE: *: Compared with the control group, *P* < 0.05 was considered of significant difference

*Abbreviations*: *EIS*, endoscopic injection sclerotherapy; *RBC*, red blood cell

**Table 4:** Rates of hospitalization and RBC transfusion, before admission and after discharge in different groups

Variables	Control group (*n* = 35)			EIS group (*n* = 31)		
	Before admission	After discharge	*P*-value	Before admission	After discharge	*P*-value
Hospitalization, *n* (%)	26 (74.29)	21 (60.00)	0.203	23 (74.19)	4 (12.90)	< 0.001^*^
RBC transfusion, *n* (%)	21 (60.00)	14 (40.00)	0.094	22 (70.97)	3 (9.68)	< 0.001^*^

NOTE: *: Compared the rates after discharge to those before admission, *P* < 0.05 was considered of significant difference

*Abbreviations*: *EIS*, endoscopic injection sclerotherapy; *RBC*, red blood cell

### Evaluation of safety of the examinations and EIS treatment

No endoscopic adverse events such as overt bleeding, small intestinal perforation, acute pancreatitis, and organ embolism occurred in the patients who underwent DBE examinations and EIS treatments until their discharges. Capsule retention occurred in none of the patients who underwent CE examinations.

### Analysis of the re-bleeding related factors

RBC transfusion before admission, multiple lesions (≥ 3), HB ≤ 70 g/L and EIS treatment were significantly associated with re-bleeding in patients with SBAs (all *P* < 0.05, Table 5). In multivariate logistic regression analysis, RBC transfusion before admission (OR, 5.655; 95% CI, 1.007–31.758, *P* = 0.049) and multiple lesions (≥ 3) (OR, 17.672; 95% CI, 2.246–139.060, *P* = 0.006) were significant risk factors of re-bleeding in patients with SBAs. In contrast, EIS treatment (OR, 0.037; 95% CI, 0.005–0.260, *P* < 0.001) was a significant protective factor (Table 6).

**Table 5:** Univariate analysis of re-bleeding related factors

Variables, *n* (%)	Re-bleeding within 12 months after discharge		OR (95% CI)	*P-*value
	Yes (*n* = 28)	No (*n* = 38)		
Age> 65 years	13 (46.43)	10 (26.32)	2.427 (0.861–6.837)	0.090
Male sex	10 (35.71)	21 (55.26)	0.450 (0.165–1.226)	0.116
History of smoking	4 (16.29)	11 (28.95)	0.409 (0.115–1.456)	0.160
History of drinking	2 (7.14)	6 (15.79)	0.410 (0.076–2.205)	0.495
Drug use of antithrombotic	4 (14.29)	4 (10.53)	1.417 (0.322–6.230)	0.935
Personal history of malignancy	2 (7.14)	3 (7.89)	0.897 (0.140–5.763)	> 0.999
History of gastrointestinal surgery	2 (7.14)	4 (10.53)	0.654 (0.111–3.348)	0.969
RBC transfusion before admission	24 (85.71)	19 (50.00)	6.000 (1.745–20.627)	0.003^*^
Hypertension	19 (67.86)	23 (60.53)	1.377 (0.494–3.840)	0.541
Liver cirrhosis	4 (14.29)	4 (10.53)	1.417 (0.322–6.230)	0.935
With type 1b lesions	23 (82.14)	25 (65.79)	2.392 (0.737–7.758)	0.140
Most lesions in jejunum	1 (3.57)	6 (15.79)	0.197 (0.022–1.744)	0.234
Multiple lesions (≥ 3)	25 (89.29)	24 (63.16)	4.861 (1.239–19.072)	0.016^*^
Lesions with active oozing/bleeding	2 (7.14)	5 (13.16)	0.508 (0.091–2.831)	0.704
HB ≤ 70 g/L	15 (53.57)	10 (26.32)	3.231 (1.147–9.102)	0.024^*^
PLT <125 10^9	7 (25.00)	9 (23.68)	1.074 (0.345–3.346)	0.902
ALB< 35 g/L	11 (39.29)	14 (36.84)	1.109 (0.406–3.030)	0.840
EIS treatment	7 (25.00)	24 (63.16)	0.194 (0.066–0.572)	0.002^*^

NOTE: We did not assess PT, APTT and TBIL because the number of patients with abnormal detection value (PT> 13.0 s, APTT> 40.0 s, or TBIL> 20.4 umol/L) < 5 in the total cohort; for the same reason, cardiovascular disease, chronic renal failure and diabetes were not involved in the univariate analysis. *: *P* < 0.05 was considered of significant difference

*Abbreviations*: *OR*, odds ratio; *CI*, confidence interval; *RBC*, red blood cell; *HB*, hemoglobin; *PLT*, blood platelet; *PT*, prothrombin time; *APTT*, activated partial thromboplastin time; *TBIL*, total bilirubin; *ALB*, serum albumin; *EIS*, endoscopic injection sclerotherapy

**Table 6:** Multivariate logistic regression analysis of re-bleeding related factors

Clinical factors	OR	95% CI	*P*-value
Age> 65 years	1.285	0.270–6.118	0.753
Male sex	0.610	0.139–2.678	0.513
RBC transfusion before admission	5.655	1.007–31.758	0.049^*^
With type 1b lesions	1.918	0.361–10.194	0.445
Multiple lesions (≥ 3)	17.672	2.246–139.060	0.006^*^
EIS treatment	0.037	0.005–0.260	< 0.001^*^

NOTE: For the multivariate logistic regression analysis, only the variables that were identified by univariate analysis as being significant with a *P*-value < 0.15 were included as covariates. RBC transfusion before admission and HB ≤ 70 g/L suggested the same clinical significance; thus, only the former was involved in the multivariate analysis. *: *P* < 0.05 was considered of significant difference

*Abbreviations*: *OR*, odds ratio; *CI*, confidence interval; *RBC*, red blood cell; *HB*, hemoglobin; *EIS*, endoscopic injection sclerotherapy

### Follow-up results

Each enrolled patient received a follow-up more than 12 months after discharge. None of the enrolled patients died within 12 months after discharge. All the patients who suffered re-bleeding were treated properly. In the control group, an 85-year-old male patient died 13 months after the discharge due to advanced age and anemia related cardiovascular accident and a 74-year-old female patient with liver cirrhosis died 48 months after the discharge due to advanced age and hepatic failure. In the EIS group, a 70-year-old male patient with liver cirrhosis and hepatocellular carcinoma died of hepatic failure 15 months after the discharge. However, there was no bleeding occurrence in this patient during the follow-up time.

### Treatments of re-bleeding after discharge

Octreotide or thalidomide was recommended for all patients with recurrent bleeding after discharge, because they refused repeated endoscopic examinations of small bowel. Considering the high cost, these patients had very low acceptance of long-term octreotide use; and they were more inclined to the temporary use of octreotide after bleeding occurrence. Among these patients, a 67-year-old female in the control group received long-acting octreotide therapy after her discharge for frequent melena. In China, the use of thalidomide in the treatment of SBAs is off-label; and it also has a lot of side effects; thus, the patient’s acceptance of long-term thalidomide use is very low. Even so, all the patients who suffered re-bleeding were treated properly in the follow-up.

In the control group, 60% of patients (21/35) experienced 1–4 times of re-bleeding and most patients required treatment of octreotide and RBC transfusion. Among the re-bleeding patients of EIS group, while 3 patients suffered 1 time of melena with spontaneous remission, the other 4 patients experienced melena or hematochezia resolved by treatment of octreotide. No new causes of bleeding were identified by examinations in these 7 patients. Most re-bleeding patients presented with chronic or intermittent bleeding and none of them experienced active massive uncontrollable hemorrhage after discharge.

## Discussion

This study showed that rates of re-bleeding, re-admission and RBC transfusion after discharge in the EIS group were significantly lower than those in the control group and the rates of hospitalization and RBC transfusion after discharge were significantly lower than those before admission in the EIS group, indicating effectiveness of EIS for treating recurrent bleeding of SBAs. RBC transfusion before admission and multiple lesions (≥ 3) were risk factors for re-bleeding and we should pay considerable emphasis to the patients with these two factors.

As the first-line investigation for detecting small bowel diseases [1, 19], CE has the capacity to achieve visualization of the entire small bowel in the overwhelming majority of patients [20]. DBE and CE have comparable diagnostic capacity in small-bowel diseases [21–24]. In spite of the advantages such as excellent safety profile, good tolerability, and low invasiveness, CE is not the primary choice of a considerable number of the patients in China, particularly relatively young patients. This condition may be due to the lack of functions as biopsy and endoscopic treatment for CE. Based on the above facts, it was reasonable that we assigned the patients diagnosed SBAs by CE or DBE to the groups of this study.

With an unclear etiology, SBAs remain challenging problems in gastroenterology. The patients with SBAs and recurrent bleeding often need to experience costly, time-consuming and complex clinical procedures. In the present study, more than half of the enrolled patients experienced hospitalization or RBC transfusion for at least once before the current hospitalization. Romagnuolo, et al. [7] reported that there were similar comparative re-bleeding rates between patients underwent endoscopic treatment and those received no therapy (42.7%, 95%CI: 38.0-47.0% versus 49.2%, 95% CI: 40.0-58.0%); and this result suggested that no less than half of the patients could not experience a recurrence, regardless of whether they received endoscopic treatment or not. For this reason, our study enrolled patients with recurrent bleeding before admission to ensure all the participants were at high risk for recurrence. To adequately evaluate the effectiveness of EIS treatment against conservative treatment, the patients who suffered bleeding at the first time were excluded in this study.

The effectiveness on conventional methods of endoscopic therapy for SBAs is still debated. The conventional techniques such as APC, contact cautery, and clips, with a pooled re-bleeding rate more than 40%, are hardly satisfactory for treating SBAs [7]. Given that the patients with SBAs are often elderly and comorbid, the endoscopists should ensure that the benefits of BAE examinations and endoscopic treatment outweigh the potential risks. The high re-bleeding rate of APC or contact cautery could be attributed to the following circumstances: the pathogenic lesions were missed; and new lesions formed in other locations after the endoscopic therapy; and the submucosal blood vessels were not cauterized enough. New therapeutic strategies for SBAs are urgently needed. With a good effectiveness and security, EIS has been used to manage various gastrointestinal vascular lesions [11–17]. However, the data on EIS for treating SBAs remains limited. Based on the mechanism of action, EIS treatment, combined with necessary clipping, can adequately occlude the pathogenic vessels. In order to reduce missed lesions as much as possible, an entire small bowel examination is necessary and the endoscopic therapy should cover all suspected lesions.

Our study employed EIS to treat recurrent bleeding of SBAs and the results showed that the mean LOS and the re-bleeding rate after discharge in the EIS group were significantly lower than those in the control group, indicating effectiveness of EIS for treating SBAs. The lower mean LOS in the EIS group also suggested shorter hemostatic process. The re-bleeding rate of patients underwent EIS treatment in this study was 22.58% (7/31), which was lower than that reported by previous studies employing conventional techniques such as APC, contact cautery, and clips [7, 9, 25]. Different inclusion criteria might lead to difference. Meanwhile, the follow-up times varied between studies; and this partly limited the significance of the comparisons. It should be noted that there were less direct studies on the independent application of hemostatic clips for the management of SBAs due to the risk of subsequent shedding; and this technique might be particularly useful to treat larger angioectasias or combine with other therapies [6]. In the control group of this study, the rate of re-bleeding was 60.00% (21/35), which was higher than that reported by the previous systematic review [7]. Our study enrolling patients with recurrent bleeding before admission may account for the difference.

In addition to the rate of re-bleeding, we also evaluated and compared the rates of re-admission and RBC transfusion after discharge and the results showed significantly lower rates in the EIS group. Meanwhile, the rates of hospitalization and RBC transfusion after discharge were significantly lower than those before admission in the EIS group; In contrast, neither the two indices had dropped significantly after discharge in the control group. Therefore, these results highlighted that EIS treatment can effectively reduce the patient’s medical needs.

Results of multivariate logistic regression analysis showed RBC transfusion before admission and multiple lesions (≥ 3) were significant risk factors of re-bleeding. Consistently, Arieira et al. founded that history of blood transfusion was associated with re-bleeding [26]. Gerson et al. [1] founded that number of the vascular lesions was a risk factor for re-bleeding of SBAs and Sakai, et al. [25] identified presence of multiple lesions (≥ 3) as the only significant independent predictor of re-bleeding. The EIS group of this study had a significantly higher proportion of patients with multiple lesions (≥ 3), which suggested the EIS group could be at higher risk in re-bleeding than the control group. Multivariate analysis also showed that EIS treatment was a significant protective factor for re-bleeding in SBAs, further indicating therapeutic value of EIS treatment.

In EIS treatment, local submucosal injection with sclerosing agent did not cause small bowel perforation in any case, even if the puncture was too deep occasionally. With a relatively low access threshold, the operation procedure of local submucosal injection is repeatable, maneuverable and time-saving. However, it is critical to limit the therapeutic range no more than 1/2 of the circumferences of intestinal wall to avoid large ulceration and delayed perforation. On the other hand, no other postoperative complications such as overt bleeding, acute pancreatitis, and organ embolism were observed in the EIS group. EIS thus had good safety.

Similar to APC and other methods, EIS cannot also completely prevent recurrent bleeding of SBAs because of the newly formed lesions or missed lesions. Supportive care, iron supplement, and RBC transfusion are still the basic treatment for patients with SBAs [1]. In addition, thalidomide and octreotide have presented some benefits [1]. Repeated endoscopic treatment may increase the effect, but also increase the risk. Thus, patients with SBAs need comprehensive treatment and continuous follow-up management to obtain the best therapeutic effect. However, considering that EIS treatment is a hemostatic method using submucosal injection directly, this technique may be more maneuverable, time-saving, secure for treating SBAs against the conventional techniques; and it may destroy deep lesions more thoroughly. EIS treatment is superior to conservative treatment; and it may be more effective than the conventional techniques including APC for treating SBAs, particularly for multiple lesions and suspected lesions.

Our study also had some limitations. Firstly, this was a single-center, retrospective case-control study. In order to obtain stronger evidence, a multicenter, prospective, randomized, double-blind clinical study with large sample and multiple endoscopic techniques is needed in the future. Secondly, the sample size involved in this study was limited because of the low prevalence of SBAs in adult patients and the strict criteria for enrollment. Thirdly, the significant differences in the age and gender distributions between groups may result in a certain amount of bias; and this may be due to the choice inclination of DBE in the relatively young male patients, given that it allows diagnosis and treatment in the same procedure. However, in this study which enrolled patients with high risk for recurrence, univariate and multivariate analysis (Tables 5 and 6), as well as the results of previous studies [25, 26], did not suggest age or gender as a significant risk factor for recurrence. Lastly, the limitations of examinations may lead to miss some small bowel lesions.

## Conclusion

EIS treatment had good effectiveness and safety for treating recurrent bleeding of SBAs, which could be considered as one of the first-line endoscopic treatment options for SBAs.

## Data Availability

The datasets used and/or analyzed during the current study are available from the corresponding author on reasonable request.

## Abbreviations

APC: Argon plasma coagulation
SBB: Small bowel bleeding
SBAs: Small bowel angioectasias
BAE: Balloon-assisted enteroscopy
DBE: Double-balloon enteroscopy
CE: Capsule endoscopy
EIS: Endoscopic injection sclerotherapy
CI: Confidence interval
OR: Odds ratio
RBC: Red blood cell
HB: Hemoglobin
PLT: Blood platelet
PT: Prothrombin time
APTT: Activated partial thromboplastin time
TBIL: Total bilirubin
ALB: Serum albumin.
