# Correction: Endoscopic injection sclerotherapy for treating recurrent bleeding of small bowel angioectasias

**DOI:** 10.1186/s12876-023-02909-x

**Published:** 2023-09-26

**Authors:** Jing Yang, Lei Zhou, Dan Xu, Yan Fan, Heng Zhang

**Affiliations:** Department of Gastroenterology, The Central Hospital of Wuhan, Tongji Medical College, Huazhong, University of Science and Technology, No.26, Shengli Street, Jiang’an District, Wuhan, Hubei Province 430014 China

**Correction: BMC Gastroenterol 23, 233 (2023)**

10.1186/s12876-023-02836-x

Following publication of the original article [1] it was reported there was an error in the author affiliation information. The correct affiliation is given in this Correction and the original article has been updated.
